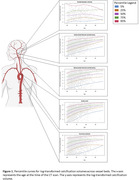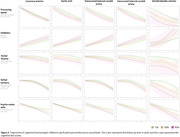# Arterial calcification in the heart‐brain axis and cognitive performance over time: a population‐based cohort study

**DOI:** 10.1002/alz.086831

**Published:** 2025-01-09

**Authors:** Anna M Streiber, Tim C van den Beukel, Ilse vom Hofe, Julia Neitzel, Meike W. Vernooij, Daniel Bos, Elisabeth J. Vinke

**Affiliations:** ^1^ Erasmus University Medical Center, Rotterdam Netherlands; ^2^ University Medical Center Utrecht, Utrecht Netherlands; ^3^ Harvard T.H. Chan School of Public Health, Boston, MA USA; ^4^ Department of Cardiovascular Sciences, Leuven Belgium

## Abstract

**Background:**

Increasing evidence shows a link between arterial calcification in the heart‐brain axis and cognitive performance. However, how calcification relates to acceleration of cognitive changes, and which specific cognitive domains are mostly affected, remains unclear. We assessed the impact of calcification in major arteries between the heart and brain on cognitive decline and focused on different cognitive domains.

**Method:**

We included 1863 participants from the population‐based Rotterdam Study (mean baseline age 67.9 ± 5.7 years, 53% female) who underwent a non‐contrast CT‐scan between 2003 and 2006 to quantify calcification in the coronary artery, aortic arch, extracranial internal carotid artery, intracranial internal carotid artery, and vertebrobasilar artery. Between 2002 and 2016, these participants also underwent two to three cognitive assessments (average follow‐up time: 9 years). The impact of arterial calcification at baseline on cognitive test performance over time was investigated using linear mixed models with a random intercept. The arterial calcification burden at baseline was transformed into age‐specific percentiles and cognition variables were log‐transformed in case of non‐normality. An interaction term was integrated between age‐specific calcification percentiles and follow‐up time, to allow for different slopes for different calcification percentiles. Non‐linearity of age and follow‐up time were taken into account by using natural cubic splines. All models were adjusted for follow‐up time, age, sex, education, APOE‐e4 carriership, and cardiovascular risk factors.

**Result:**

The age‐specific percentile curves of arterial calcification for each artery are presented in Figure 1. We found that a higher calcification burden was associated with worse baseline cognitive performance across various domains, specifically psycho‐motor functioning. Figure 2 highlights the cognitive trajectories in different arteries, showing that cognition declines faster in participants with higher calcification volumes across all cognitive domains. Importantly, the effect of calcification on cognitive performance differs across the arteries. Both intercept differences and accelerated cognitive decline seem most pronounced for calcification in the vertebrobasilar arteries.

**Conclusion:**

A larger amount of arterial calcification in the heart‐brain axis, is robustly associated with accelerated cognitive decline over time, specifically in the psychomotor domain. Our findings suggest that arterial calcification in arteries closer to the brain exerts most influence on cognitive decline.